# Mechanical properties and progressive failure characteristics of sandstone containing elliptical and square openings subjected to biaxial stress

**DOI:** 10.1371/journal.pone.0246815

**Published:** 2021-03-04

**Authors:** Honggang Zhao, Haitao Sun, Dongming Zhang, Chao Liu

**Affiliations:** 1 State Key Laboratory of Coal Mine Disaster Dynamics and Control, Chongqing University, Chongqing, China; 2 State Key Laboratory of the Gas Disaster Detecting, Preventing and Emergency Controlling, Chongqing, China; 3 China Coal Technology and Engineering Group Chongqing Research Institute, Chongqing, China; 4 School of Mechanics and Civil Engineering, China University of Mining and Technology, Xuzhou, China; China University of Mining and Technology, CHINA

## Abstract

Two kinds of common tunnel shapes, i.e. elliptical opening and square opening were selected for biaxial compression tests, and the influences of two kinds of opening shapes on the mechanical properties, failure characteristics and failure modes of sandstone were compared and analyzed. The complex variable theory and mapping functions were used to obtain the analytical stress solution around elliptical and square openings. The results show that the stability of the specimen containing an elliptical opening was better than that of the specimen containing a square opening under the same lateral stress. Compared with the elliptical opening, the local damage was formed earlier in the square opening which might be caused by a higher stress concentration around the square opening. The stress distributions around openings were influenced by the opening shape and lateral stress coefficient. The top and bottom of square opening were more prone to tensile fracture, and the distribution range of tensile was larger than that of elliptical opening. When the opening failed, the intensity of square opening failure was weaker than that of elliptical opening. On the basis of the average frequency value and the rise angle value, the failure mode of specimen containing elliptical or square opening was distinguished. It was found that the mixed tension and shear failure dominated the failure of specimens with different opening shapes, and the number of shear cracks in the specimen containing a square opening was greater than that in the specimen containing an elliptical opening. The above method of judging failure mode by acoustic emission signals was well verified by the CT images of damaged specimens.

## Introduction

As the mining depth gradually increases and the development of underground excavation engineering, the studies on the stability of tunnels are more and more extensive and in-depth. After entering the deep underground excavation engineering, the tunnel excavation results in the stress redistribution around the opening [1–3]. The radial stress turns to zero at the boundary of tunnel, while the compressive tangential stress gradually increases. When this high compressive tangential stress reaches or exceeds the strength of rock, the rock will be destroyed [4]. These failures are often manifested as spalling at sidewalls, tensile fracture in the top and bottom, and even rockburst [3]. They threaten the safety of workers and cause equipment damage, even damage to the underground structure.

A series of studies have been carried out to investigate the mechanical and failure characteristics around tunnels by means of laboratory experiments and numerical simulations [5–12]. As a common shape of the tunnel opening, the failure and instability process of circular tunnel has been widely studied. Under the polyaxial compression, the evolution of fractures around holes was studied by Lajtai, et al. [13], and the results suggested that the tensile mode dominates the cavities at a low confining pressure, while the position of the compressive stress concentration caused a large deformation under a higher confining pressure. Fakhimi, et al. [5] used the sandstone specimen containing a circular hole to simulate the failure process and the PFC^2D^ was applied to simulate the failure zone which was observed in the physical experiment. The finite element code RFPA2D was used to analyze the failure process around the circular opening. Results showed that the tensile cracks dominated the failure process under a low confining pressure, while the initiation and propagation of tensile cracks were restrained under a higher confining pressure and the failure process was dominated by shear cracks [14]. Yang, et al. [15] studied the variation of instantaneous stress during the excavation process and a theoretical model suitable for 2D circular excavation was established. Liu, et al. [16] investigate the effect of water contents on the stability of tunnel. They found that more AE events were generated in saturated tunnel model and the saturated tunnel model was more damaged in the early loading stage.

However, the shape of tunnel is not only circular, but also elliptical, square, inverted U-shaped and so on. The influence of opening shape on the mechanics, failure characteristics, stress concentration and fracture patterns of rocks has been demonstrated [17–23]. The fracturing processes around the circular, elliptical and inverted U-shaped openings were shown by using RFPA [17] and the influence of lateral pressure coefficients were considered. Liu, et al. [24] conducted the uniaxial compressive test and studied the spatial-temporal evolution of micro-cracks in the specimen with an inverted U-shaped hole, and the results showed that the failure of hole was dominated by shear cracks. Li, et al. [20] analyzed the fracturing process and deformation around the elliptical opening and found that the variation of the strain localization zones has a significant effect on the propagation of tensile cracks around the opening. However, few reports have been published that focused on the comparisons of influences of different opening shapes on rock mechanical properties, failure characteristics and failure mode, especially on comparisons of stress distribution around the different opening shapes.

In this paper, the elliptical opening and square opening were drilled in sandstone specimens. The mechanics properties, failure characteristics and failure modes of specimens were investigated under biaxial compression. The tangential stress distribution around different opening shapes was analyzed and some suggestions were put forward for the support of tunnels with elliptical and square openings.

## Experimental methodology

### Specimen description and preparation

**Fig 1:**
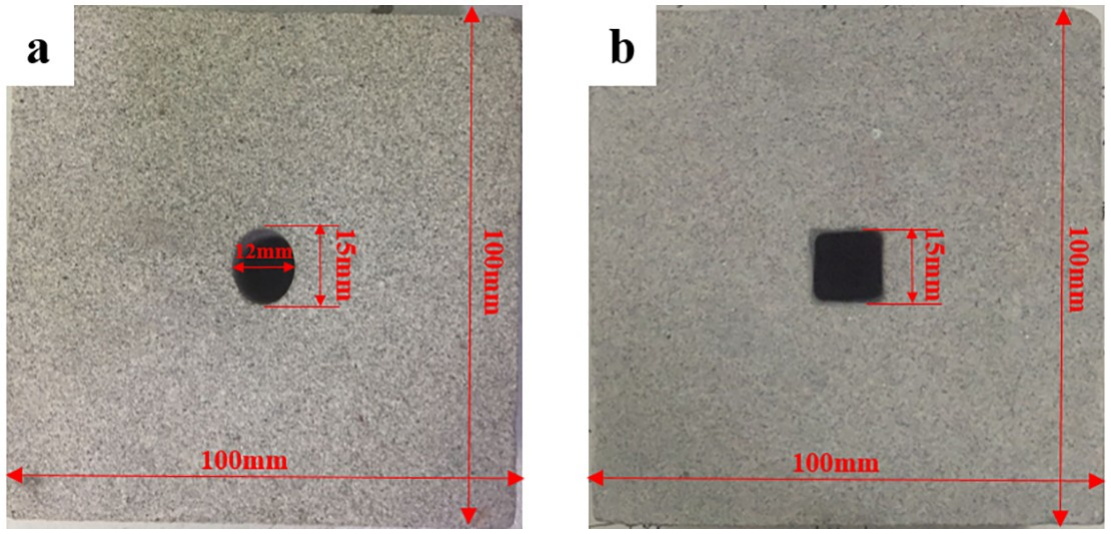
The prepared specimens with different opening shapes. (a) elliptical; (b) square.

Sandstone was used for the tests in this study. The sandstone is grayish white, homogeneous, and isotropic. The basic physical parameters of sandstone are listed in Table 1. Firstly, the sandstone specimens were obtained from a large block rock mass to reduce discreteness. Then, they were cut and burnished as rectangular prisms, and the size of specimens is 100 mm×100 mm×50 mm in width, height, and thickness, respectively. Finally, two common tunnel cross-section shapes were machined at the center of specimens. The size of ellipse is 15 mm × 12 mm in longitudinal axis and transverse axis, respectively, and the side length of square is 15 mm. The specimens used in this study are shown in Fig 1.

**Table 1:** Basic physical parameters of sandstone.

Specimen	UCS/MPa	Tensile strength/MPa	E/GPa	*v*
**Sandstone**	79.09	6.86	5.64	0.22

### Experimental equipment and procedure

**Fig 2:**
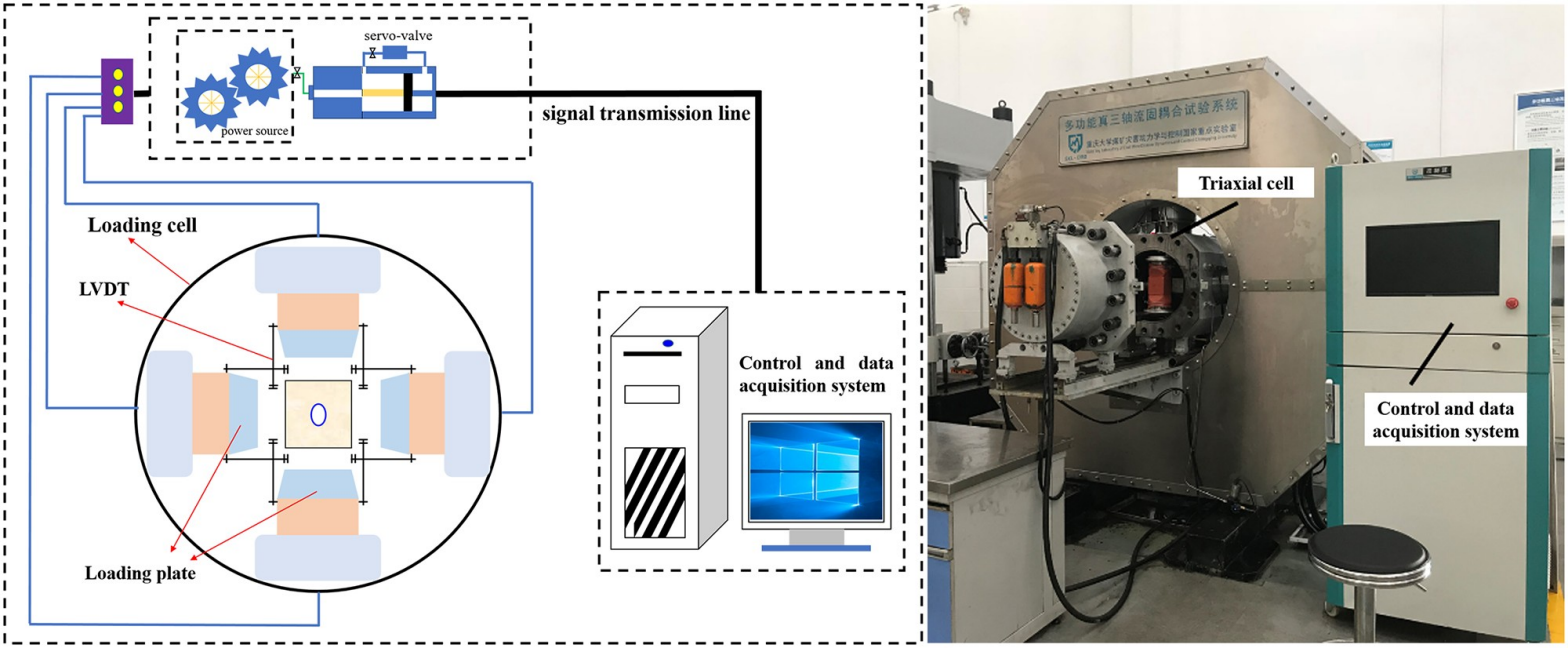
Multifunctional true triaxial geophysical apparatus.

A newly developed multifunctional true triaxial (TTG) apparatus was applied to conduct the biaxial compression tests in this study [23, 25]. A maximum force of 6000 kN in two directions can be loaded to the rock specimen and the maximum force which can be applied in another direction is 4000 kN. There are six loading heads in the triaxial cell of equipment, and each loading head can be controlled independently. It can realize the simulation of different excavation stress paths. The detailed introduction of this equipment can be referred to Li, et al. [26]. The schematic diagram and physical drawing of the equipment are shown in Fig 2. In addition, the acoustic emission (AE) monitoring system was used to collect the AE signals during the entire experimental process, and the failure processes of openings was captured by a high-speed camera.

According to the previous study, there is a limited depth of rockburst in surrounding rock of tunnel. The state of biaxial compression and true triaxial compression within the limited depth can both lead to the instability of tunnel [27, 28]. In order to conveniently observe the failure process of opening, the biaxial stress compression testes were designed and the phenomenon of failure during the process of stress adjustment of elliptical and square opening after excavation was simulated. First, the desired vertical stress *σ*_v_ and lateral stress *σ*_h_ were loaded to 20 MPa simultaneously. Then, *σ*_h_ was kept unchanged and *σ*_v_ was continually loaded at a displacement rate of 0.002 mm/s until the sandstone specimen was destroyed.

## Results and analysis

### AE characteristics and the strength of sandstone specimens with different opening shapes

**Fig 3:**
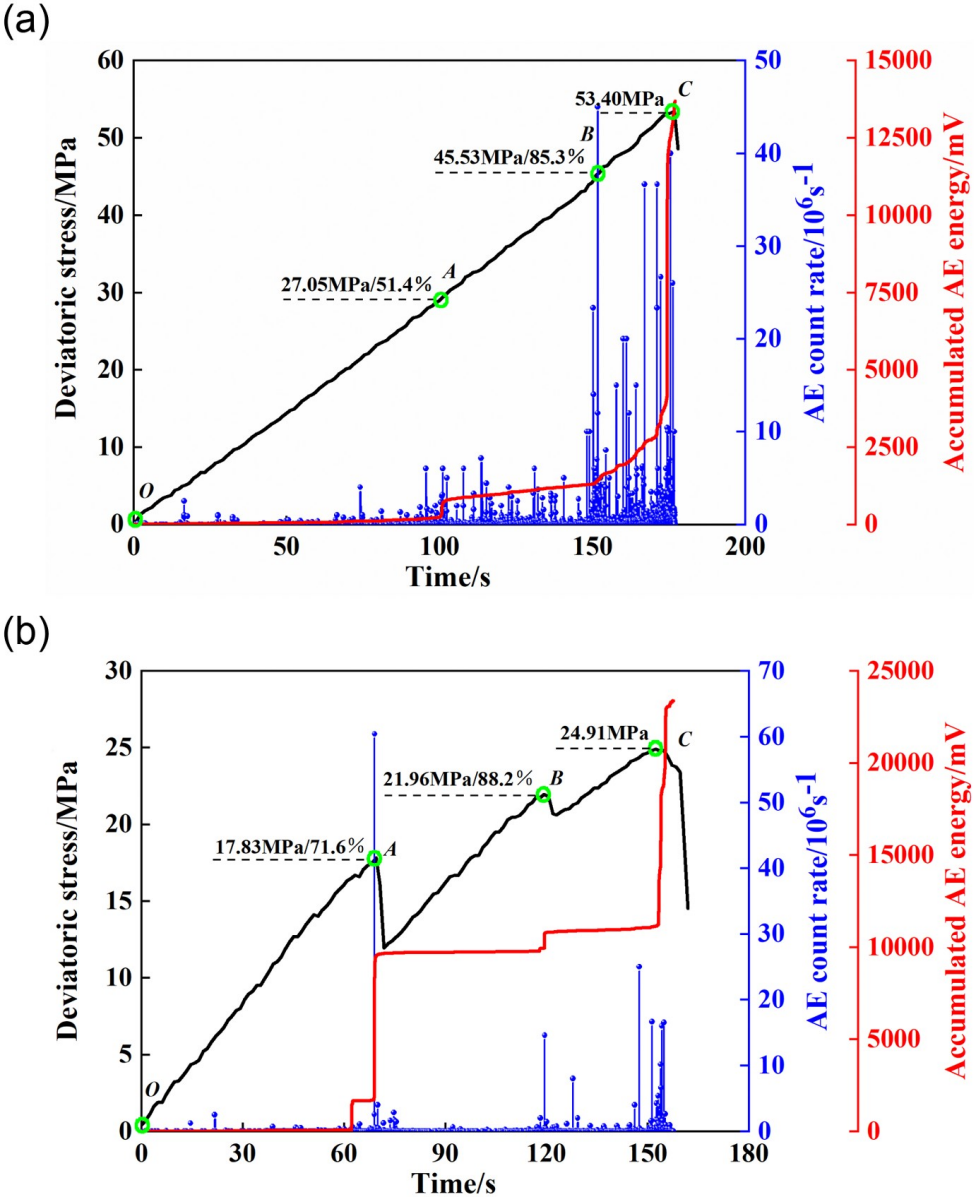
Deviatoric stress curves associated with AE count rate and accumulated AE energy variations for different opening shapes. (a) elliptical opening specimen; (b) square opening specimen.

Fig 3 presents the deviatoric stress (*σ*_v_-*σ*_h_) with the AE count rate and accumulated AE energy variation curves. For the specimen containing an elliptical opening, it exhibited an elastic behavior during the whole loading process. When (*σ*_v_-*σ*_h_) = 27.45 MPa, the AE events became active and the cumulative AE energy sharply increased (Point *A*), which indicated that the micro-cracks began to initiate and propagate steadily. When (*σ*_v_-*σ*_h_) = 45.53 MPa, a high AE count rate appeared and the cumulative AE energy curve climbed sharply from point *B* (Phase *BC*). It implied that the cracks entered the stage of unstable growth, and finally formed macro fractures in this period [29]. Moreover, due to that the sharp increase of the AE energy only occurred when the specimen containing an elliptical opening was close to failure, thus, we can predict the instability of elliptical tunnel according to these AE characteristics.

For the specimen containing a square opening, there were several stress drops during the failure process and the first stress drop occurred when (*σ*_v_-*σ*_h_) = 17.83 MPa. This phenomenon was related to the initiation of cracks. Compared with the elliptical opening, the local damage was formed earlier in the square opening which might be caused by a higher stress concentration around the square opening. After point *A*, the AE events were inactive and the cumulative AE energy-time curve entered a platform stage, which implied that the elastic strain energy was reaccumulated until the overall failure occurred. Although a slight decrease of the (*σ*_v_-*σ*_h_) occurred at point *B*, the magnitude of the increase of the cumulative AE energy was relatively small, which indicated that the degree of crack damage in point *B* was low. When the (*σ*_v_-*σ*_h_) reached the peak stress (point *C*), the AE count rate was relatively small compared with that of the elliptical opening, which may be attributed to that vast amounts of cumulative elastic strain energy consumed by the local fracture in specimen containing a square opening before reaching the peak strength. Therefore, when the overall failure of tunnel occurred, kinetic energy transformed from elastic strain energy would be reduced and thus abated the intensity of the failure. In addition, from Fig 3, we can observe that the (*σ*_v_-*σ*_h_)_max_ of elliptical opening was significantly greater than that of square opening, which implies that the elliptical opening can alleviate the concentration of high stress around the opening and improve the stability of tunnel.

### Tangential stress distribution around the openings

The instability of tunnel closely related to the stress distribution around the opening. The equation of tangential stresses at the boundary of elliptical opening was derived by Timoshenko, et al. [30]. However, the stress distribution at different distances from the boundary of the opening is still unclear. Moreover, the scope of the failure after tunnel excavation is not only limited near the boundary of the opening, but also causes different degrees of damage in the deep surrounding rock. Thus, it is necessary to study the tangential stress distribution around the openings and even far from the openings that contributes to better understandings of the failure mechanism of tunnel. In our study, MATLAB was applied to derive analytic solutions for the stress fields around the elliptical opening and square opening by employing a complex function and a conformal transformation as the bridge, and these functions can be degenerated into the classical stress field equations of a circular opening.

#### Analytical solutions of stress around the opening

**Fig 4:**
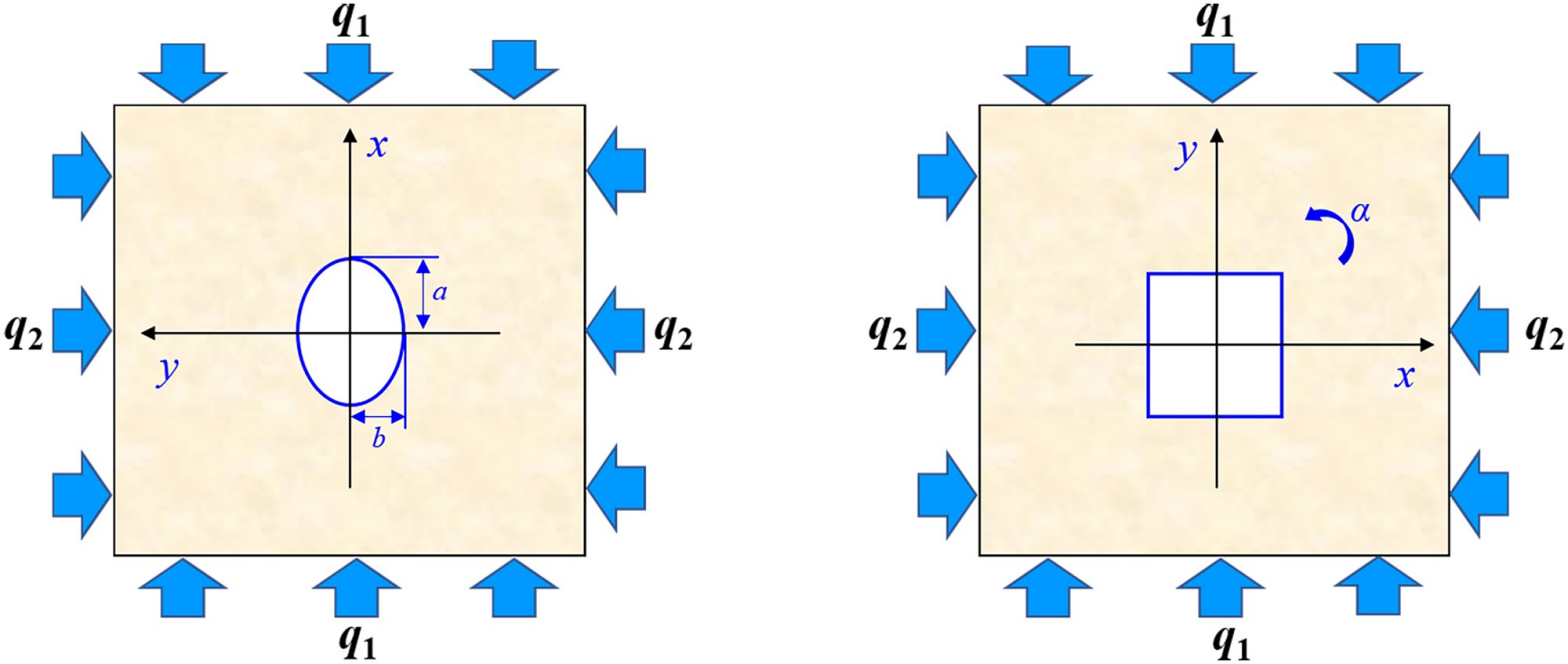
Schematic diagram of the stress boundary of an elliptical opening and square opening under far-field stress.

In this study, sandstone, which is suitably based on its characteristics (i.e., isotropic, continuous, homogeneous and elastic body), was selected for the experiments. To obtain the analytic solutions, the boundary conditions for the elliptical opening and square opening were first determined as follows (Fig 4):

$$\sigma_{1}=-q_{1}$$ (1)

$$\sigma_{2}=-q_{2}$$ (2)

The real constants related to the far-field stress are:

$$B=\frac{\sigma_{1}+\sigma_{2}}{4}=-\frac{q_{1}+q_{2}}{4}$$ (3)

$$B\prime +\text{i}C\prime =-\frac{1}{2}(\sigma_{1}-\sigma_{2})e^{-2\text{i}\alpha }=\frac{1}{2}(q_{1}-q_{2})e^{-2\text{i}\alpha }$$ (4)

where *σ*_1_ and *σ*_2_ are principal stresses parallel to the cross section of the opening (MPa). *q*_1_ and *q*_2_ represent the magnitudes of in situ stress (MPa). *B*, *B*’ and *C*’ are given real constants characterizing the remote stress field. *B* is proportional to the sum of the two principal stresses at infinity in the elastic body, and *B*’ + i*C*’ is proportional to the difference of the two principal stresses at infinity in the elastic body (MPa). *i* and *a* express an imaginary unit and the angle between the X-axis direction and the maximum principal stress, respectively.

For openings with different shapes, the region occupied by an object on one complex plane can be mapped to the interior or exterior of the central unit circle on another complex plane by conformal mapping [31, 32]. Thus, we use the following mapping functions to simplify the geometry of the elliptical opening (Eq 5) and square opening (Eq 7).

$$z=\omega (\xi )=R(\xi +\frac{m}{\xi })$$ (5)

m=(a−b)/(a+b) (6)

$$z=\omega (\xi )=R(\xi -\frac{1}{6}\xi^{-3})$$ (7)

Eqs 5 and 7 show that the points z on the complex plane Z occupied by an ellipse and square that are mapped to the interior of the central unit circle on the complex plane *ξ*. Here, *ξ* is the point on the complex plane *ξ*. *R* is the shape parameter of elliptical opening and square opening. *a* and *b* are the semiaxes of the ellipse, and 0 ≤ *m* ≤ 1.

For the purpose of facilitating the calculation in the complex function solution, *f*_0_ is introduced [31] and the expression is as follows:

$$f_{0}=i\int \left(\bar{f_{x}}+i\bar{f_{y}}\right)ds-\frac{f_{x}+if_{y}}{2\pi }\text{ln}\;\sigma -\frac{1+\mu }{8\pi }(\bar{f_{x}}-i\bar{f_{y}})\frac{\omega (\sigma )}{\bar{\omega^{\prime }(\sigma )}}\sigma -2B\omega (\sigma )-(B^{\prime }-iC^{\prime })\bar{\omega (\sigma )}$$ (8)

where $\bar{f_{x}}$ and $\bar{f_{y}}$ are surface forces of the microunit at the boundary of opening. *f*_*x*_ and *f*_*y*_ is the principal vector of the surface force. Since the supporting force on the opening surface does not exist, the values of *f*_*x*_, *f*_*y*_, $\bar{f_{x}}$ and $\bar{f_{y}}$ are equal to 0. *u* is Poisson’s ratio.

The single valued analytic functions *φ*_0_(ξ) and *ψ*_0_(*ξ*) are associated with *f*_0_ and $\bar{f_{0}}$. The equation of *φ*_0_(ξ) and *ψ*_0_(*ξ*) of elliptical and square opening can be expressed as follows:

$$\left\{ \begin{array}{l} \varphi_{0}(\xi )=\frac{1}{2\pi i}\int_{\sigma }\frac{f_{0}}{\sigma -\xi }d\sigma \\ \psi_{0}(\xi )=\frac{1}{2\pi i}\int_{\sigma }\frac{\bar{f_{0}}}{\sigma -\xi }d\sigma -\xi \frac{\xi^{2}+m}{m\xi^{2}-1}\varphi_{0}^{\prime }(\xi ) \end{array}\right.$$ (9)

$$\left\{ \begin{array}{l} \varphi_{0}(\xi )=\frac{1}{2\pi \text{i}}\int_{\gamma }\frac{\omega (\sigma )}{\bar{\omega^{\prime }(\sigma )}}\frac{\bar{\varphi_{0}^{\prime }(\sigma )}}{\sigma -\xi }\text{d}\sigma -\frac{1}{2\pi \text{i}}\int_{\gamma }\frac{f_{0}\text{d}\sigma }{\sigma -\xi }\\ \psi_{0}(\xi )=\frac{1}{2\pi \text{i}}\int_{\gamma }\frac{\bar{\omega (\sigma )}}{\omega^{\prime }(\sigma )}\frac{\varphi_{0}^{\prime }(\sigma )}{\sigma -\xi }\text{d}\sigma -\frac{1}{2\pi \text{i}}\int_{\gamma }\frac{\bar{f_{0}}}{\sigma -\xi }\text{d}\sigma \end{array}\right.$$ (10)

where Eq (9) denotes the elliptical opening and Eq (10) denotes the square opening. *φ*_0_(ξ) and *ψ*_0_(*ξ*) are related to the complex functions *φ*(*ξ*) and *ψ*(*ξ*), which are the two analytical function represented by the complex function *ξ* and can be used to characterize the stress function in the plane problem where the body force is a constant.

$$\left\{ \begin{array}{l} \varphi (\xi )=-\frac{1+\mu }{8\pi }(f_{x}+if_{y})\text{ln}\;\xi +B\omega (\xi )+\varphi_{0}(\xi )\\ \psi (\xi )=\frac{3-\mu }{8\pi }(f_{x}-if_{y})\;\text{ln}\;\xi +(B^{\prime }+iC^{\prime })\omega (\xi )+\psi_{0}(\xi ) \end{array}\right.$$ (11)

Incorporating Eqs (3)–(5), (7) and (8) into Eqs (9) and (10), we can obtain the single valued analytic functions *φ*_0_(ξ) and *ψ*_0_(*ξ*). Then, according to the Eq (11), the complex functions *φ*(*ξ*) and *ψ*(*ξ*) can be obtained as follows:

$$\left\{ \begin{array}{l} \varphi (\xi )=R[\frac{q_{1}+q_{2}}{4}m\xi +\frac{q_{2}-q_{1}}{2}\xi e^{2i\alpha }-\frac{q_{1}+q_{2}}{4}\frac{1}{\xi }]\\ \psi (\xi )=R\{\xi [\frac{q_{1}+q_{2}}{2}(1-m\frac{\xi^{2}+m}{m\xi^{2}-1})-\frac{q_{2}-q_{1}}{2}\frac{\xi^{2}+m}{m\xi^{2}-1}e^{2\text{i}\alpha }]-\frac{q_{2}-q_{1}}{2}\frac{1}{\xi }e^{-2\text{i}\alpha }\} \end{array}\right.$$ (12)

$$\left\{ \begin{array}{l} \varphi (\xi )=R[-\frac{q_{1}+q_{2}}{4}\xi -(q_{1}-q_{2})(\frac{3}{7}\text{cos}\;2\alpha +\frac{3}{5}\text{i}\;\text{sin}\;2\alpha )\xi^{-1}-\frac{\left(q_{1}+q_{2}\right)}{24}\xi^{-3}]\\ \psi (\xi )=R[\frac{1}{2}(q_{1}-q_{2})e^{-2\text{i}\alpha }\xi -\frac{13}{6}(q_{1}-q_{2})(\frac{3}{7}\text{cos}\;2\alpha +\frac{3}{5}\text{i}\;\text{sin}\;2\alpha )\frac{\xi }{2\xi^{4}+1}+\frac{13}{12}(q_{1}+q_{2})\frac{\xi^{3}}{\left(2\xi^{4}+1\right)}] \end{array}\right.$$ (13)

where *α* is the angle between the X-axis direction and the maximum principal stress. Eq (12) denotes the elliptical opening and Eq (13) denotes the square opening.

Thus, the stress field around the elliptical opening and square opening can be obtained through the following equation.

$$\Phi (\xi )=\frac{\varphi^{\prime }(\xi )}{\omega^{\prime }(\xi )}$$ (14)

$$\Psi (\xi )=\frac{\psi^{\prime }(\xi )}{\omega^{\prime }(\xi )}$$ (15)

$$\sigma_{r}+\sigma_{\theta }=4\text{Re}[\Phi (\xi )]$$ (16)

$$\sigma_{\theta }-\sigma_{r}+2\text{i}\tau_{r\theta }=\frac{2\xi^{2}}{\rho^{2}\bar{\omega^{\prime }(\xi )}}[\bar{\omega (\xi )}\cdot \Phi^{\prime }(\xi )+\omega^{\prime }(\xi )\Psi (\xi )]$$ (17)

where *σ*_*r*_ is the radial stress (MPa), *σ*_*θ*_ is the tangential stress (MPa), and *τ*_*rθ*_ is the tangential shear stress (MPa). *Φ*(*ξ*) and *Ψ*(*ξ*) are the two analytical functions represented by the complex function *ξ*. Re represents the real part of the complex function *Φ*(*ξ*). Since the above formulas are too long and the amount of calculation is large. Thus, the MATLAB programming was used to calculate the analytical solutions quickly and accurately for stress.

**Fig 5:**
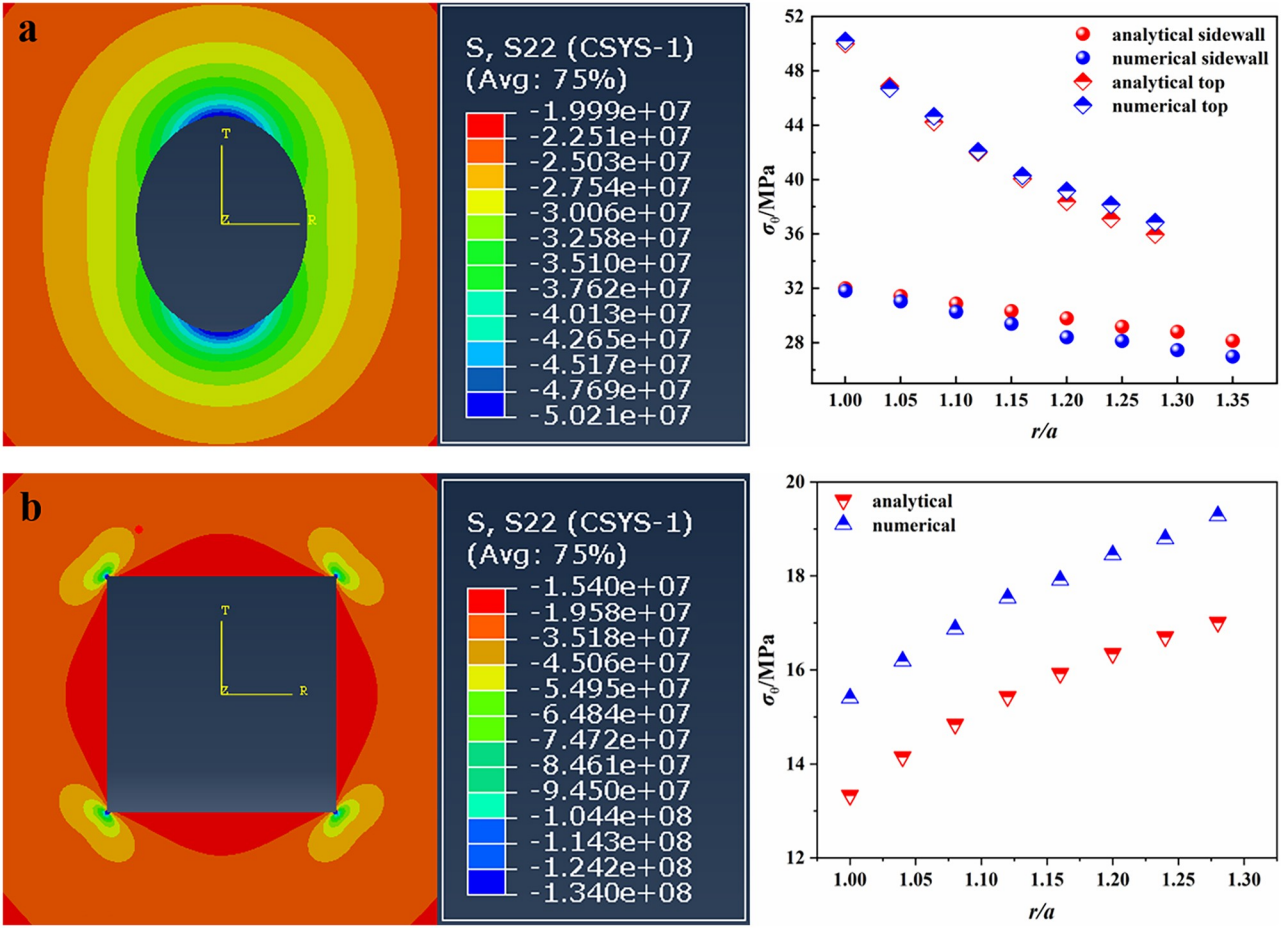
Local stress nephograms and comparison results. (a) elliptical opening; (b) square opening.

Before analyzing the stress distribution around the opening with the above analytical solutions of stress, we verified the accuracy of analytical solutions by using the numerical simulation software Abaqus. In our simulation, the plane strain was assumed. The model sizes were set to the same proportions as the physical models. A total of 241271 quadrilateral elements and 245780 quadrilateral elements were generated in the model with elliptical opening and the model with square opening, respectively. Considering that the biaxial compression tests were conducted in our study, the boundary conditions of model containing elliptical or square opening were set as that the vertical far field stress was equal to horizontal far field stress and equal to 20 MPa (lateral stress coefficient λ = 1). In order to avoid the plastic deformation, the young’s modulus of two models was set larger enough [33]. Other required parameters were consistent with the actual values of sandstone specimen. Fig 5 showed the local stress nephogram around the opening and the comparison results between the theoretical and numerical methods. It should be noted that when λ = 1, the tangential stress *σ*_*θ*_ on the top and bottom of square opening was the same as that on the sidewall of square opening. Thus, only the tangential stresses *σ*_*θ*_ on the top of square opening were listed in Fig 5b. From Fig 5, we can observe that for the sandstone specimen containing an elliptical opening, the tangential stresses *σ*_*θ*_ obtained by two methods were very similar (Fig 5a). However, for the sandstone specimen containing a square opening, there was a relatively big difference between the results obtained by the two methods, and the difference between the two results was about 2 MPa. Furthermore, the tangential stresses *σ*_*θ*_ at the corners of square opening calculated by theoretical and numerical methods were 120 MPa and 134 MPa, respectively. The above differences may be related to the size of quadrilateral elements. The smaller the element size, the more accurate the numerical results [33]. In addition, in numerical simulation, the corner of square opening is 90°, while the corner of square obtained by mapping function transformation is circular arc, which can reduce the stress concentration. Thus, the theoretical result is smaller than the numerical result. However, the trend of theoretical result is consistent with that of numerical result and the magnitude of them is the same. Therefore, the theoretical result can correctly reflect the tangential stress distribution around the square opening.

#### Analysis of the tangential stress distribution around the openings

**Fig 6:**
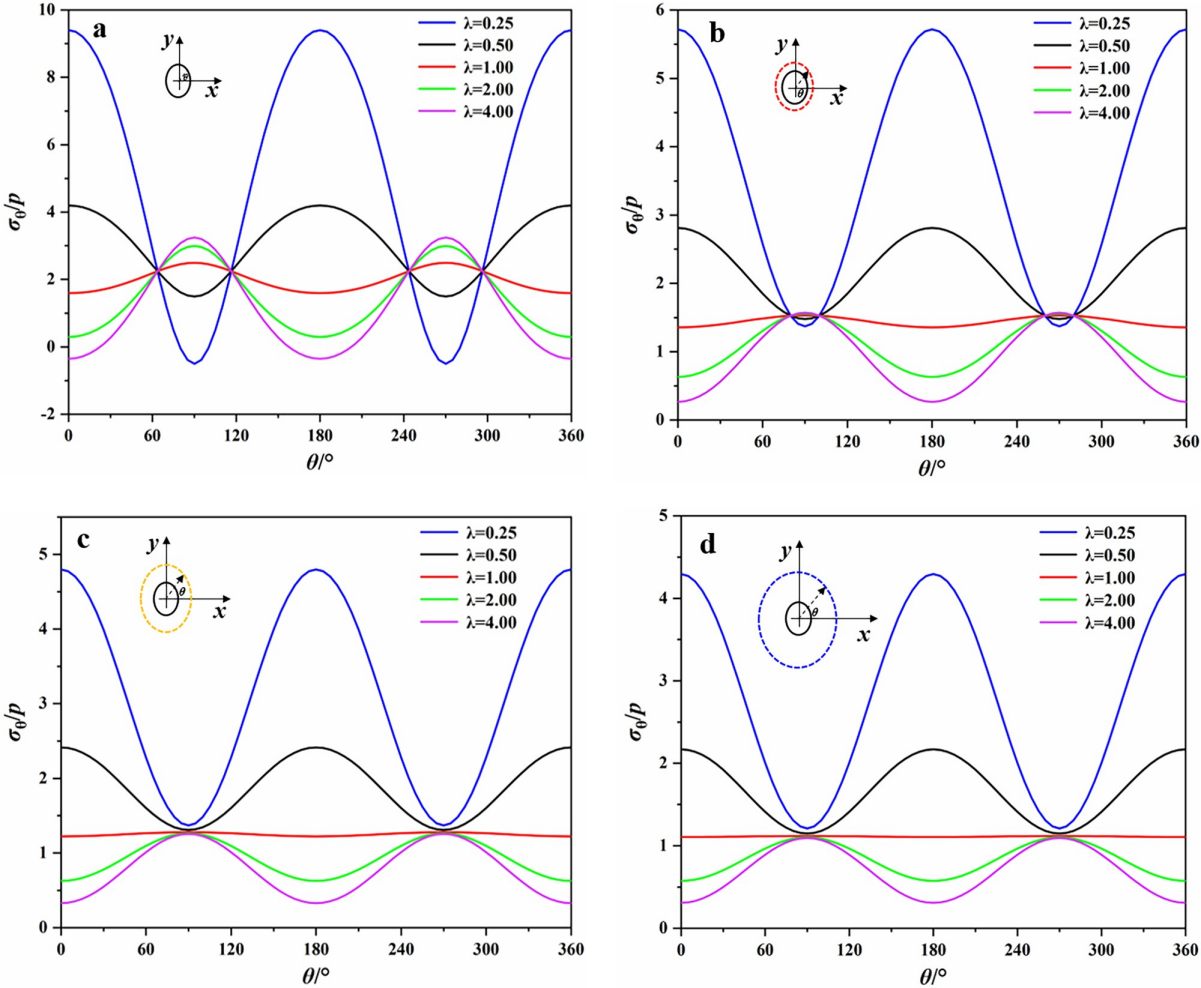
Stresses at different distances from the boundary of elliptical opening. (a) boundary; (b) 1.5 times; (c) 2 times; (d) 3 times.

**Fig 7:**
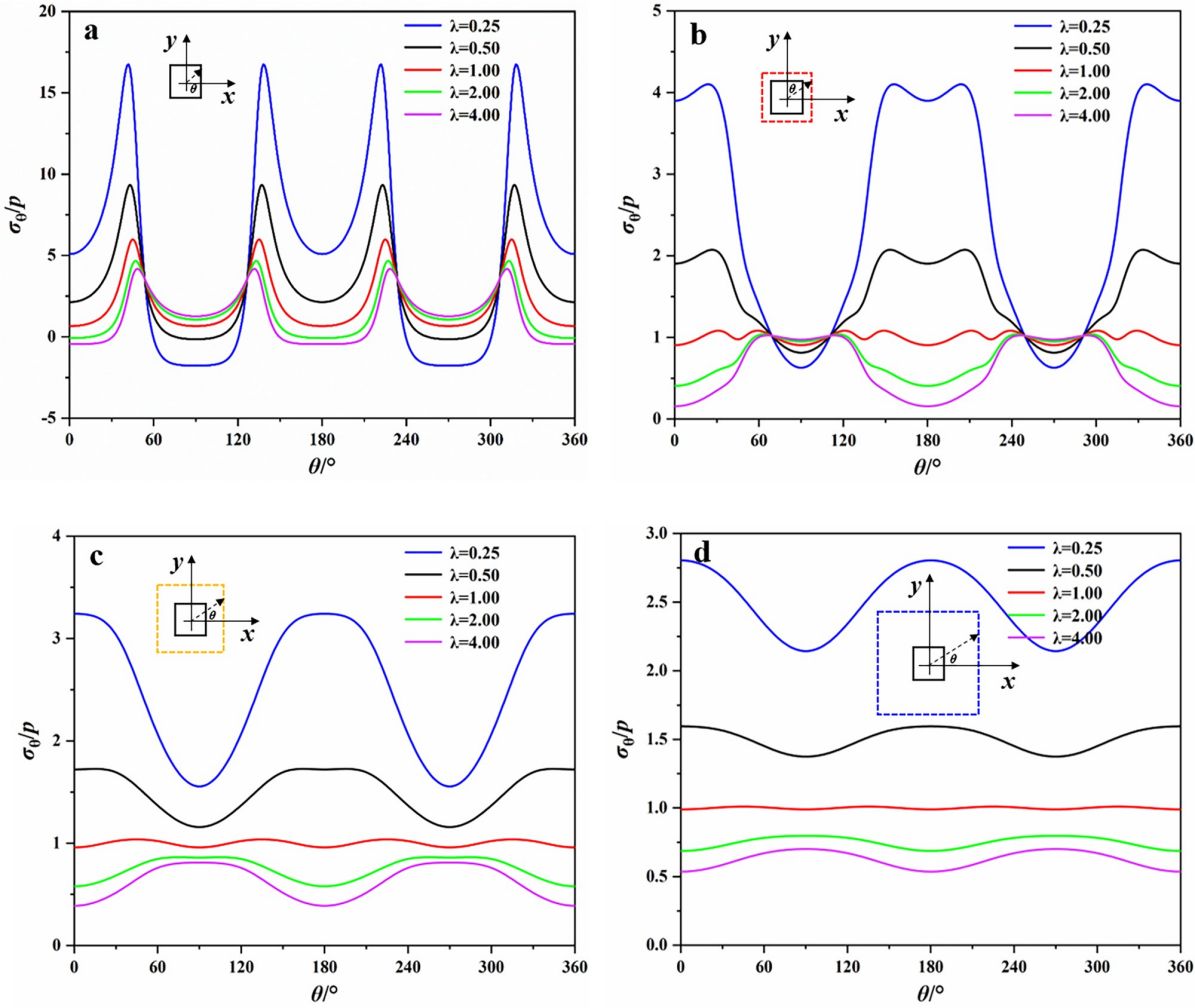
Tangential stresses at different distances from the boundary of square opening. (a) boundary; (b) 1.5 times; (c) 2 times; (d) 3 times.

Figs 6 and 7 presented the tangential stress distribution around the opening with different lateral stress coefficients λ at the boundary and distances of 1.5, 2 and 3 times from the opening center. *θ* in these figures was measured counterclockwise from the x-axis and varies from 0° to 360°. *p* in Figs 6 and 7 represents the lateral stress, which is equal to 20 MPa in our study. In Figs 6 and 7, the positive value represents the tangential compressive stress and the negative value represents the tangential tensile stress. As was shown in Figs 6 and 7, the distribution of *σ*_*θ*_ around the elliptical opening was obviously different from that of the square opening. For the boundary of elliptical opening, when λ = 0.25, the tensile stress concentrated on the top and bottom of opening. The maximum compressive stress concentrated on the two sidewalls of opening and the maximum compressive stress concentration coefficient was 9.40. With the increase of λ, the tangential stresses on the top and bottom gradually changed from tensile stress to compressive stress, and the opposite situation occurred on the sidewalls. For example, when λ = 1, the maximum compressive stress appeared on the top and bottom. And when λ = 4, the tensile stress appeared on the sidewalls. With the increase of the distance from the boundary of elliptical opening, the surrounding rock was basically subjected to compressive stresses and the value of them gradually decreased.

For the boundary of square opening, the maximum compressive stress always concentrated near the corners of opening and the maximum compressive stress concentration coefficient was 16.76 (λ = 0.25). It implies that there is a high probability of failure at the corners where the support is required. When λ = 0.25, the tensile stress concentrated on the top and bottom. As λ increases, the change of tensile stresses on the top and bottom of square opening was similar to that of elliptical opening. When λ = 2, the tensile stresses began to appear at the sidewalls. With the increase of the distance from the boundary of square opening, the curve of tangential stresses distribution around square opening was gradually similar to that around elliptical opening, and the maximum compressive stress shifted from the corners to the sidewalls, which suggests that the damage caused by the compressive stress gradually shifts from the corners to the sidewalls as the distance increases.

**Fig 8:**
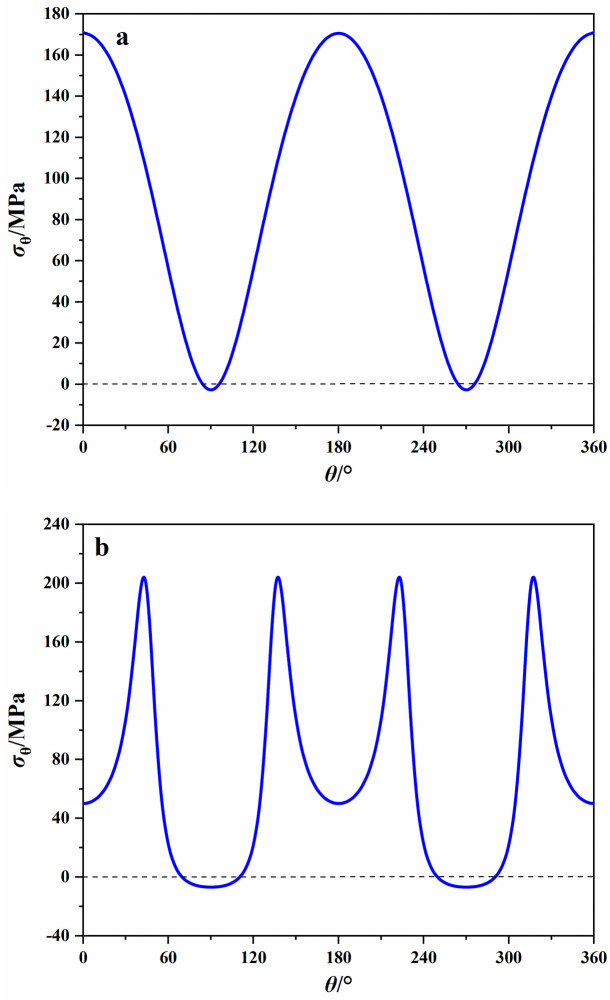
Tangential stresses at the boundary of openings when the loading stress reaches the peak stress. (a) elliptical opening; (b) square opening.

Based on the analytical stress solution and test results, we can calculate the tangential stress *σ*_*θ*_ at the boundary of openings at the time of rock failure and the stress distribution curves were shown in Fig 8. As was shown in Fig 8a, when the vertical stress reached the peak stress, the tensile stress zone was found on the top and bottom of elliptical opening and the maximum tensile stress was -2.87 MPa, which was smaller than the uniaxial tensile strength of sandstone. Thus, the tensile cracks may not be formed on the top and bottom. This is consistent with the results of numerical simulation by Wang, et al. [14] that a higher confining pressure restrains the initiation and propagation of tensile cracks. The maximum compressive stress at the middle of sidewall was 170.87 MPa, which was far greater than the uniaxial compressive strength. This led to the formation of slabbing fractures induced by splitting tension at the boundary of sidewall [33].

For the specimen containing a square opening (Fig 8b), when the vertical stress reached the peak stress, the maximum tensile stress on the middle of top and bottom was -6.82 MPa. It was almost equal to the uniaxial tensile strength of sandstone. This implied that tensile cracks were more likely produced on the top and bottom of square opening compared with the elliptical opening. The maximum compressive stress at the corner of square opening was 204.64 MPa, which means that the rock at the corner has been damaged before rock failure.

**Fig 9:**
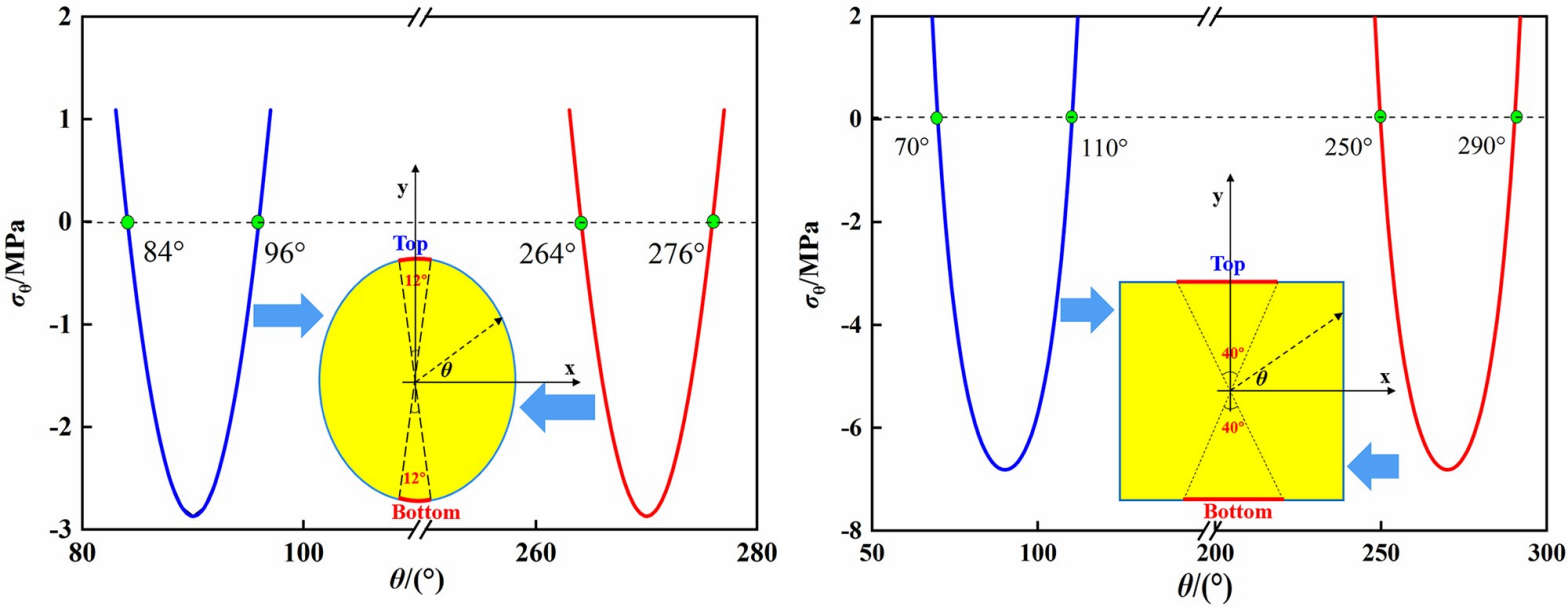
Regions of tensile stress at the boundaries of the elliptical and square openings.

For the actual underground excavation engineering, the above results imply that in order to prevent and control the rock fracturing caused by high compressive stress concentration, the support in the middle of the sidewalls should be strengthened in the elliptical tunnel, and the support at the corner of the square tunnel should be strengthened or apply localized rounding to reduce the stress concentration [21]. In addition, under the same lateral stress, the tensile stress at the top and bottom of the square opening is larger than that of the elliptical opening, which is more likely to cause the tensile fracture. Hence, the support at the top and bottom of square opening should be strengthened compared with the elliptical opening. Moreover, due to that the tensile strength of rock is far less than the compressive strength, the rock may be damaged under a small tensile stress. Thus, we not only need to pay attention to the magnitude of the tensile stress, but also need to study the distribution range of the tensile stress, so as to reasonably support. Based on the analytical solutions of stress fields around the openings, the region of tangential tensile stress on the top and bottom of different opening shapes can be calculated, as shown in Fig 9. It can be seen that the tensile stress zones on the top and bottom of the elliptical opening are distributed between 84° and 96°, 264° and 276°, respectively. For the square opening, the tensile stress zones on the top and bottom are distributed between 70° and 110°, 250° and 290°, respectively. As a result, the tensile fracturing regions on the top and bottom of square opening is larger than those of elliptical opening.

### Analysis of the failure process of the openings

#### Characteristics analysis during the failure process

**Fig 10:**
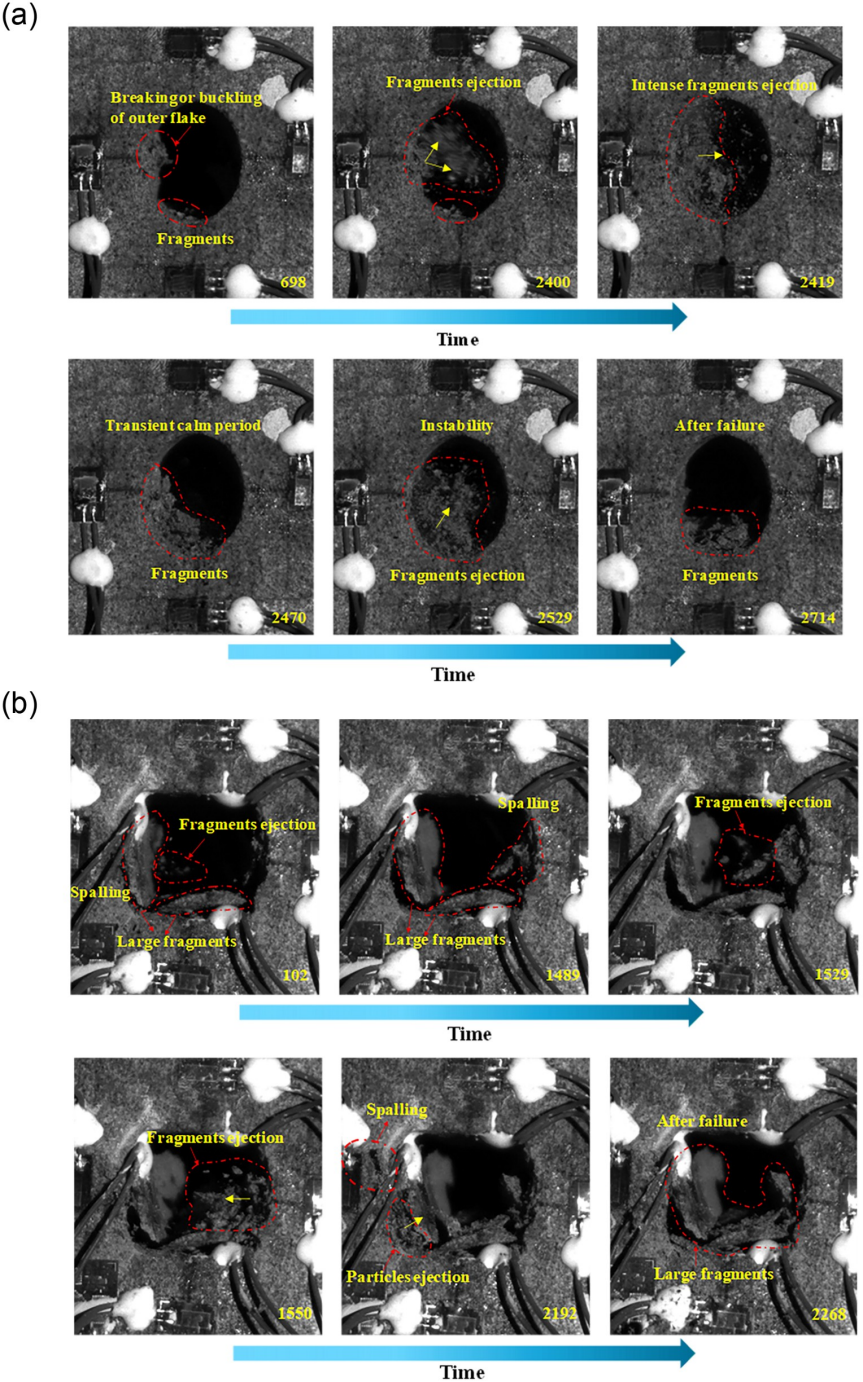
Typical failure phenomena of specimens with different opening shapes recorded using a high-speed camera. (a) elliptical opening specimen; (b) square opening specimen.

Fig 10 shows the failure process of elliptical and square opening recorded by the high-speed camera. Since the working time of high-speed camera used in our study is 4 seconds and the focus in this section is the failure process of the openings. Thus, the changing process of the opening during the early loading phase was not recorded. For the elliptical opening (Fig 10a), at 698 ms, clear spalling and some fragments could be observed at left sidewall and bottom, respectively. This phenomenon was induced by the development of extensile cracks subparallel to the opening boundary. When the growth of these extensile cracks proceeds up to at least the point of creating thin rock plates of buckling size, the thin rock plates break and separate from the rock wall. After spalling, the continuous ejection of particles occurred and particles could be found on the bottom. As the vertical stress gradually increased, the first intense fragments ejection occurred at approximately 2400 ms with a large amount of fragments erupting, which is named unstable or violent failure [34], and the failure at this stage was obviously violent compared with the spalling. At this moment, the residual strain energy after fracturing was transformed into kinetic energy and released, which led to a relatively large ejection velocity of fragments. After the first rockburst, which lasted for approximately 70 ms, a new relatively stable equilibrium state was achieved and lasted for 59 ms. Then, a second intense fragments ejection occurred at the same position, resulting in deeper and wider failure region. In addition, the dimensions of fragments were relatively small, which may be caused by the large kinetic energy.

For the square opening (Fig 10b), during the initial moments recorded by the high-speed camera (i.e., 102 ms), two thick rock plates have been separated from the sidewall and some relatively thin rock plates that were not completely separated from the right sidewall appeared. This situation may be caused by the local failure, which was induced by the high compressive stress concentration near the corners of the square opening during the early loading stage. With the increase in the vertical stress, some fragments continued to drop out from the sidewall. At about 1489 ms, the obvious spalling failure began to appear at the right sidewall, and was accompanied by the ejection of relatively large fragments. This process lasted for approximately 61 ms and then achieved a relatively stable equilibrium state. At 2192 ms, a large number of particles ejected from the corner into the opening space. Due to that the high compressive stress mainly distributed around the corner, it is reasonable to speculate that the shear failure occurred in the corner at this time [20]. From the failure process of the square opening, it can be seen that the whole sidewall was damaged, resulting in a larger-sized rock plate, and then the dimension of fragments gradually decreased. Thus, the shape of square opening changed to a hexagon. In addition, the failure of square opening is relatively peaceful compared with that of the elliptical opening. This may be caused by the release of accumulated strain energy in the square opening during the loading, thus reducing the kinetic energy when the overall failure occurred. This agrees with the characteristics of AE signals analyzed in section 3.1.

#### Fracture patterns of different opening shapes

Generally, shear fracture, tensile fracture and mixed shear fracture and tensile fracture are the common fracture modes during rock failure, and we can identify these fracture modes depending on the AE signals induced by the initiation and propagation of different types of cracks [35]. Thus, the ratio of the average frequency value (i.e., AF) to the rise angle value (i.e., RA) was proposed to identify the failure modes [23, 34, 36, 37].

**Fig 11:**
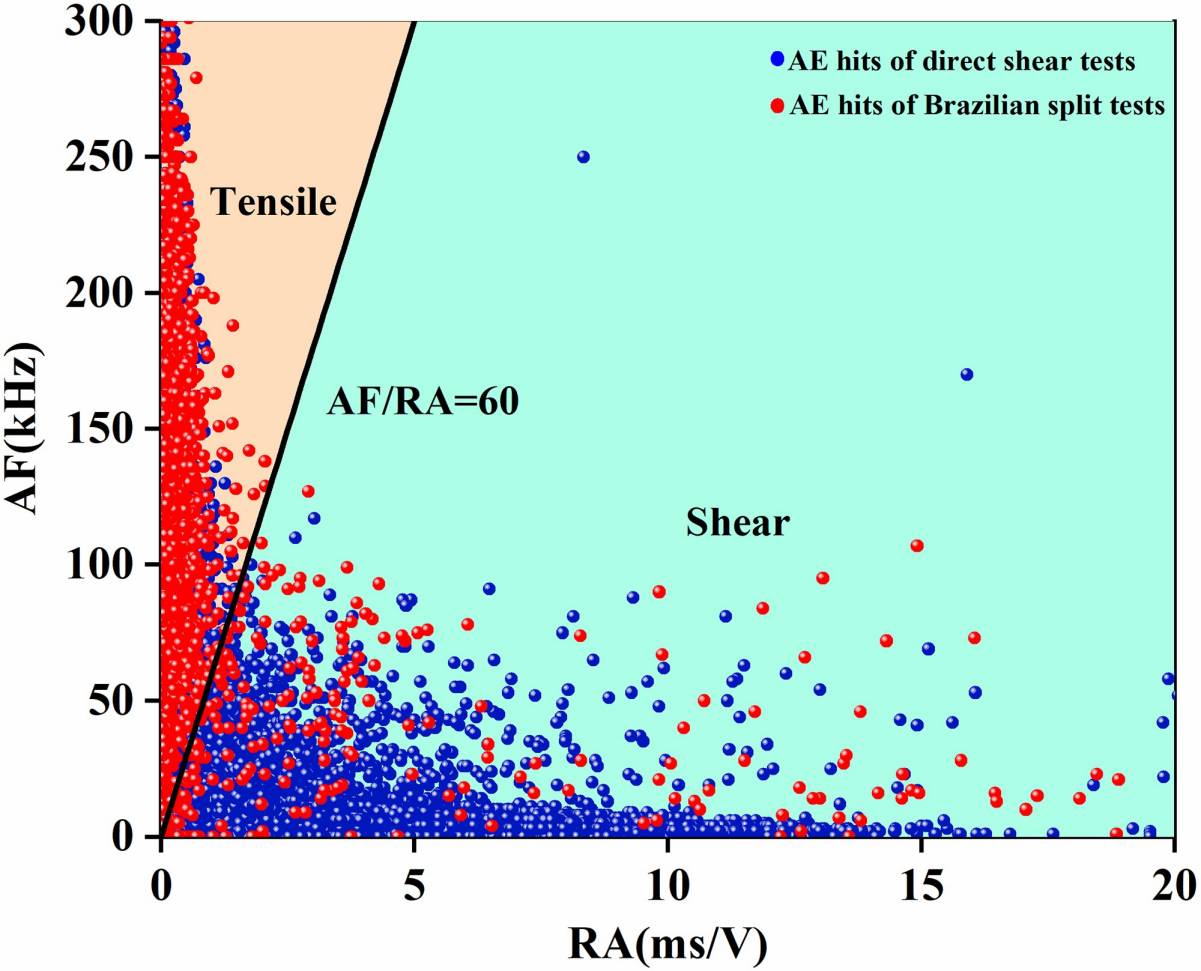
Scatter plot of AF and RA.

**Fig 12:**
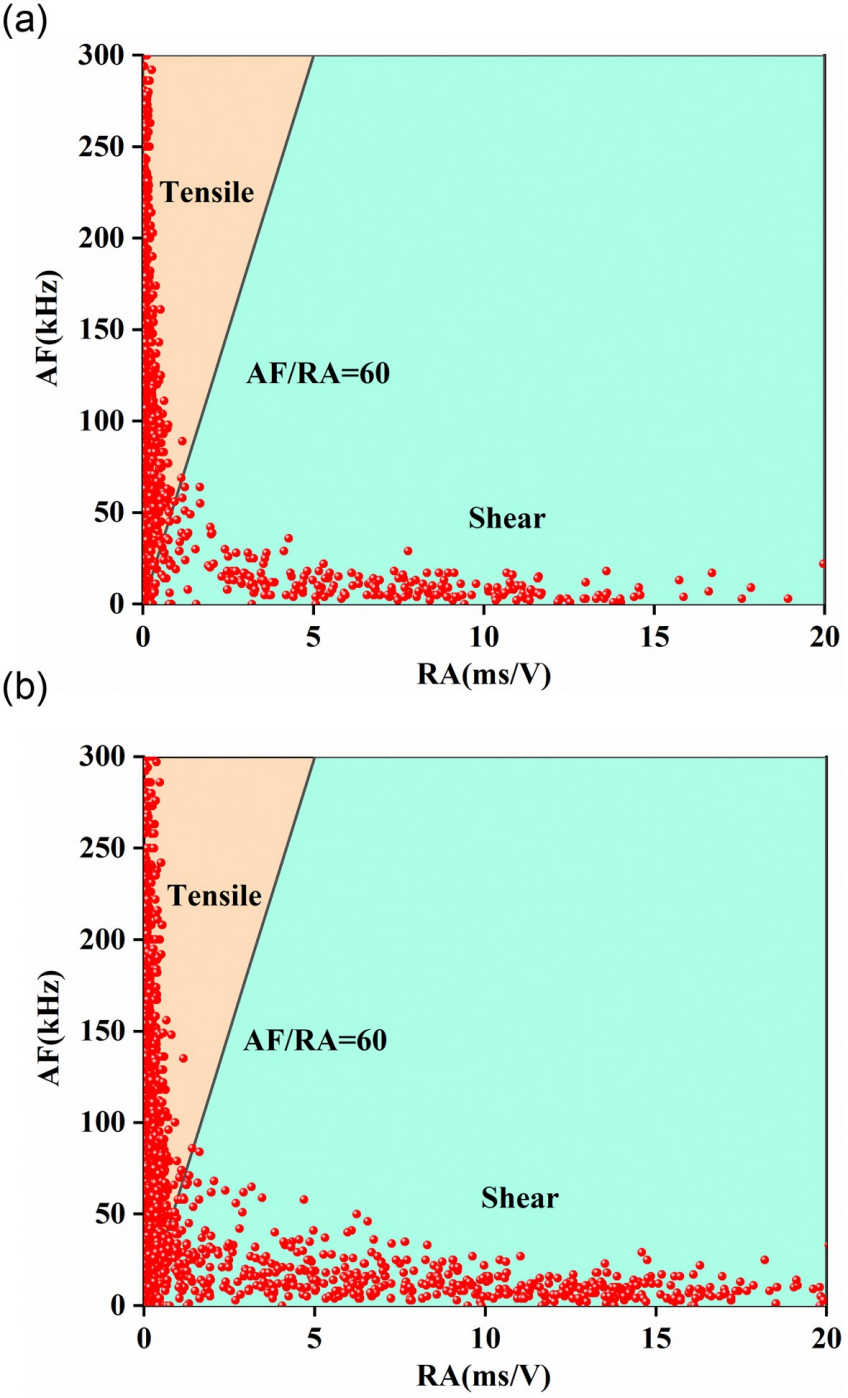
Scatter plots of the AF and RA with different opening shapes. (a) elliptical opening specimen; (b) square opening specimen.

According to the published results [23], we obtained the accurate value of AF/RA used to characterize the tensile fractures and shear fractures by conducting the Brazilian split tests and direct shear tests. Based on the test results, the value of AF/RA was defined as 60 to distinguish the mode of fractures. From Fig 11, we can observe that the AE signals representing the tensile crack are basically distributed above the curve representing AF/RA = 60, and the AE signals below the curve indicate the shear crack. The scatterplots of the AF and RA values of sandstone specimens with different opening shapes obtained under biaxial compression are shown in Fig 12. We can see that in the region which the values of AF/RA were less than 60, the density of AE signals of the square opening (Fig 12b) was significantly greater than that of the elliptical opening (Fig 12a). According to the calculated values of AF/RA, we were able to determine that the ratios of shear signals during the failure processes of elliptical opening and square opening were 7.2% and 22.0%, respectively. In summary, the above results suggest that under the biaxial compression, the failure modes of specimens both express mixed tension and shear failure, and the number of shear cracks in the specimen containing a square opening is greater than that in the specimen containing an elliptical opening.

**Fig 13:**
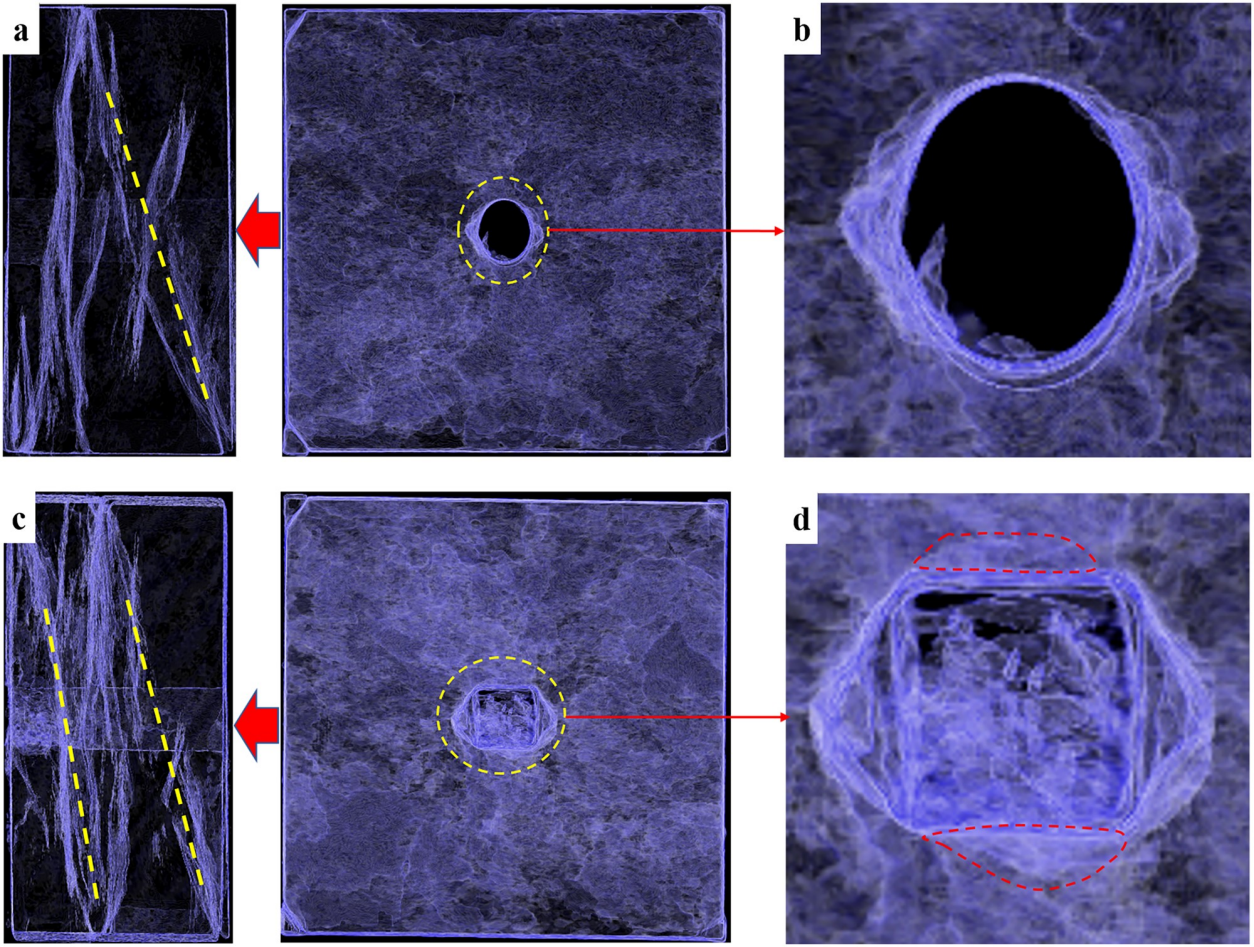
Images of CT scanning. (a) left view of the elliptical opening; (b) front view of the elliptical opening; (c) left view of the square opening; (d) front view of the square opening.

**Fig 14:**
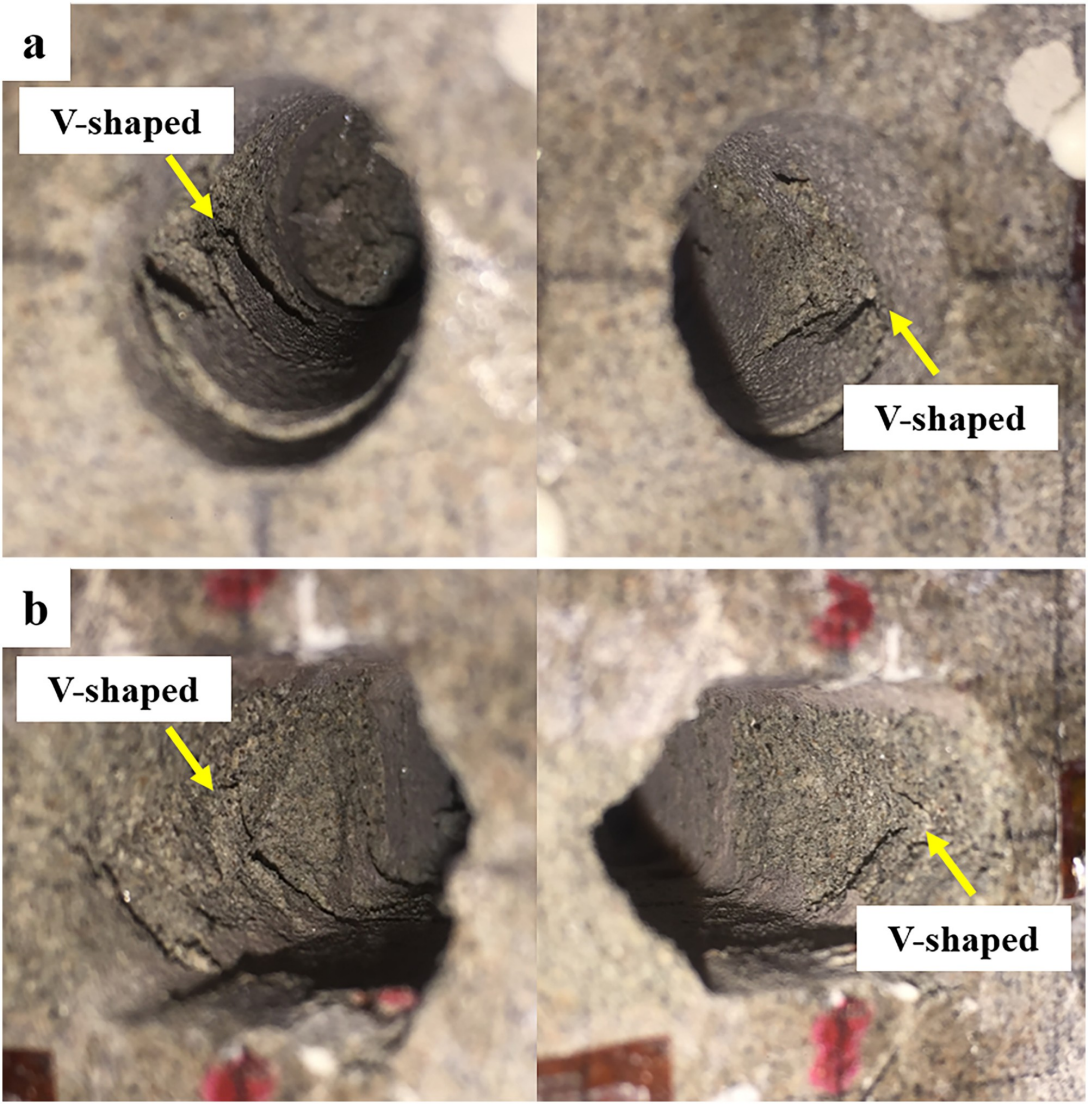
V-shaped failure bands after specimen failure. (a) elliptical opening; (b) square opening.

By carrying out the CT scanning on the failure specimens, we obtained the left views and front views of the elliptical opening and square opening (Fig 13). It can be found that the left views (Fig 13a) showed that only one shear sliding band was formed in the specimen containing an elliptical opening, while two parallel shear sliding bands were formed in the specimen containing a square opening (Fig 13c). These results are consistent with the results of the AE signals analysis. In addition, on the top and bottom of elliptical opening (Fig 13b), cracks cannot be observed, while some micro-cracks were found on the top and bottom of square opening, as shown in the red dotted line in Fig 13d. This phenomenon was associated with the distribution and magnitude of tensile stress in elliptical and square openings, which was analyzed in section 3.2.2. Fig 14 shows the V-shaped failure bands located at the sidewalls after specimen failure. Combined with Figs 13 and 14, we can also find that the damage region of the elliptical opening was mainly symmetrically concentrated on the middle part of sidewall and was approximately triangular, while the sidewall of square opening was damaged as a whole and the most destructive area was located in the middle of sidewall. As a result, the shape of the opening changed from square to hexagonal after specimen failure, which is consistent with the results of numerical simulation by Feng, et al. [38]. It indicates that the whole sidewall of square tunnel should be supported, while the support at the middle part of sidewall needs to be strengthened in the elliptical tunnel.

## Conclusions

In order to investigate the effect of opening shape on rock mechanics, stress distribution, failure process and fracture patterns, elliptical and square holes were machined at the center of sandstone specimens. The biaxial compression tests were carried out. The AE system and a high-speed camera were also applied to record the AE signals and capture the failure process of openings, respectively. The main conclusions are as follows:

1. The opening shape has a significant effect on the mechanical properties of specimen. The compressive strength of specimen containing a square opening is significantly greater than that of the specimen containing an elliptical opening. Several stress drops which indicates the formation of local fractures appear in the specimen containing a square opening during the process of stress adjustment after tunnel excavation. According to the characteristics of AE energy, it provides the evidence that the local fractures can effectively consume the cumulative elastic strain energy.
2. According to the complex function and mapping functions, the analytical solutions of tangential stress around the elliptical and square openings are established. When the specimen is subjected to the biaxial compression, the tensile stress on the top and bottom of square opening is larger than that of elliptical, which indicates that it is easier to form tensile cracks on the top and bottom in square opening compared with elliptical opening. This well corresponds to the results observed in CT images of specimens. In addition, the tensile regions on the top and bottom of square opening is larger than those of elliptical opening.
3. According to the values of AF and RA, the failure modes of opening specimens are distinguished. The results show that the under biaxial compression, the failure modes of specimens containing elliptical or square opening both express mixed tension and shear failure, and the number of shear cracks in the specimen containing a square opening is greater than that in the specimen containing an elliptical opening. The above results are well verified by the CT images of damaged specimens.

## Supporting information

S1 Fig Documents on proof of funds. (PDF)

S1 Data Origin data. (XLSX)
