# Supplementary material for: Mechanical properties and progressive failure characteristics of sandstone containing elliptical and square openings subjected to biaxial stress
Source: PLoS One. 2021 Mar 4;16(3):e0246815. doi: 10.1371/journal.pone.0246815 (PMC7932157; doi:10.1371/journal.pone.0246815)
Supplement: S1 Fig — (PDF) [file pone.0246815.s001.pdf]

2019年重庆大学
“重庆市研究生科研创新项目”任务书

2019 Chongqing University

“Graduate Student Research Innovation Project” Mandate

Honggang Zhao

姓　　名（Name）：赵宏刚

学　　号（Student ID）：20182001045

学　　院（College）：资源及环境科学学院

学　　科（Subject）：矿业工程

项目类型（Project Type）：博士学术类

项目编号（Project Number）：CYB19045

项目基金号（Fund Number）：CYB19045

重庆大学研究生创新实践基地

第七条　项目负责人发表的专著、公开发表学术论文（含学位论文）、研究报告等研究成果必须在基金**前三位标注**重庆大学“重庆市研究生科研创新项目”资助，项目基金号：CYB19045 或在致谢加注“Project supported by graduate research and innovation foundation of Chongqing, China (Grant No. CYB19045)”，结题验收成果将对负责人和成员分别审核。项目完成取得成果的知识产权归研究生院与项目负责人双方所有。**（注意：同一研究成果不能用于本项目多个项目结题）**

三、项目经费的拨付及使用

第八条　研究生院对项目负责人资助金额为：人民币 壹万 元整(大写)，¥ 10000 元（小写），实报实销　1500 USD

第九条　如若项目负责人未在项目完成时间前，按本任务书项目成果规定完成研究任务，取得预期成果，研究生院有权追回所有资助经费，并依据重庆大学及研究生院相关规定做出处理。

第十条　项目负责人应对项目经费专款专用，不得挪作他用。

第十一条　经费使用范围：科研业务费、实验材料费、仪器设备费等相关经费、日常公用经费、固定资产构建和修缮及自筹基建等所需费用。不能用于**劳务费、人员经费报销**。

第十二条　本项目经费的管理和使用严格按照《重庆大学“重庆市研究生科研创新项目”资金管理办法》的规定执行。

四、项目实施注意事项

第十三条　项目负责人所申报的“创新项目”开展过程中如果需要与病毒或有毒、有害物质接触，或是涉及有危险的操作、试验等，项目负责人必须严格杜

第十八条 若项目不能按时结题，项目负责人可向研究生院提交延期申请，其期限不能超过一年，经批准同意后方可延期。但须在毕业答辩前一个月完成项目结题。

第十九条 成果转让和转化过程中涉及的知识产权及相应权益等问题，遵照国家法律法规和学校有关规定执行。

第二十条 本任务书解释权归重庆大学研究生院所有。

重庆大学研究生院

2019年9月17日

Dear Editor (s),

Title: Mechanical properties and progressive failure characteristics of sandstone containing elliptical and square openings subjected to biaxial stress

Authors: Honggang Zhao, Haitao Sun, Dongming Zhang, Chao Liu

I am honored to be able to submit our paper to PLOS ONE.

We notice that there is a charge for the publication of the manuscript. The publication fees appear too expensive for me at this moment. Although this project was supported by the Graduate Research and Innovation Foundation of Chongqing (CYB19045), the total amount of the grant was RMB10000 (about 1500 USD). With this amount, we could just mange to conduct and complete our experiments, and thus we do not have enough funding to pay for the publication fees.

Therefore, I am writing you to explore the possibility that the publication fees could be reduced. If you have a difficulty to offer us such a concession, we will make our every effort to meet the requirements for the publication including payment of the publication fees since the publication of this paper in PLOS ONE is very important for us.

Documents on proof of funds are included in the supporting material and have been uploaded.

Thank you very much for your consideration, and look forward to hearing from you soon.

With kind regards, and wish you a merry Christmas in advance

Yours sincerely

Haitao Sun

Corresponding author

China Coal Technology and Engineering Group Chongqing Research Institute, Chongqing 400030, China

E-mail: dreamsht@163.com

Honggang Zhao Haitao Sun

Dongming Zhang Chao Liu
